# Mixed Cryoglobulinemia in a Patient with Juvenile Idiopathic Arthritis

**DOI:** 10.1155/2019/5858106

**Published:** 2019-06-06

**Authors:** Fahad Bnatig, Leen Raddaoui, Talal Hijji, Lina El Kibbi

**Affiliations:** ^1^College of Medicine, Alfaisal University, Riyadh, Saudi Arabia; ^2^Department of Internal Medicine, George Washington University Hospital, Washington, DC, USA; ^3^Department of Rheumatology, Specialized Medical Center Hospital, Riyadh, Saudi Arabia

## Abstract

Cryoglobulinemia is a rare illness of cryoglobulin accumulation in the blood which can typically present with arthralgia, purpura, skin ulcers, glomerulonephritis, and peripheral neuropathy. It is classified as mixed cryoglobulinemia when cryoglobulins contain more than one immune component such as IgM rheumatoid factor and polyclonal IgG. Typically, it presents in the setting of clonal hematologic disease, viral infection, or certain connective tissue diseases. Herein, we report the case of a 24-year-old man diagnosed and treated as mixed cryoglobulinemia in the setting of juvenile idiopathic arthritis (JIA). Investigations for viral etiologies, including HBV, HCV, and HIV, and all serologic tests were negative. Additionally serum protein and urine protein electrophoresis did not reveal monoclonal gammopathy; however, testing for plasma cryoglobulins was positive, and qualitative analysis revealed a faint polyclonal pattern. Thus, he was diagnosed with cryoglobulinemia in the setting of JIA, which has not been reported in the literature before. He dramatically improved upon initiation of rituximab and methotrexate.

## 1. Introduction

Cryoglobulinemia is a rare multisystem disease characterized by the presence of circulating immunoglobulins that precipitate upon exposure to cold temperatures, and can manifest clinically by the triad of purpura, weakness, and arthralgias [1]. Mixed cryoglobulinemia (types II and III) is characterized by variable organ involvement, for which the clinical manifestations can be wide-ranging. It can also be associated with numerous infections, immunological diseases, and certain malignancies [2]. For this reason, patients with mixed cryoglobulinemia pose a unique diagnostic and therapeutic challenge for physicians.

## 2. Case Report

A 26-year-old middle-eastern man presented with dusky discoloration of his skin involving his upper and lower extremities for 4 years. The discoloration involved the dorsal surfaces of his metacarpals and metatarsals, which appeared only upon exposure to cold temperatures, associated with mild discomfort, and disappeared with rewarming initially. Upon presentation, the discoloration was visible at room temperature.

Upon further questioning, he was found to have been diagnosed with juvenile idiopathic arthritis since childhood. During his childhood, he had stiffness upon elbow extension, which was more pronounced on the right side than on the left, as well as stiffness upon movement of his knees. In the past, he also complained of morning stiffness lasting for a few minutes as well as stiffness after sitting for prolonged periods of inactivity which improves throughout the day. Additionally, he had mild periodic swelling of finger joints, but not accompanied by pain or redness. At the present presentation, he denied having fever, joint stiffness or redness, pain, and joint swelling. He also denied weight changes, muscle weakness or tenderness, fatigue, hair loss, visual disturbances, eye or mouth dryness, mouth ulcers, numbness/weakness of the lower extremities, and skin rash. His family history was remarkable for juvenile idiopathic arthritis in both his mother and sister. He was started on iron supplementation since childhood and oral prednisone at age 16 to manage his juvenile idiopathic arthritis; otherwise, he took no other medications. Review of systems was negative for any other complaints, and he denied any history of surgical procedures.

On examination, he was sitting comfortably in no apparent distress, with no pallor of the conjunctiva or lymphadenopathy of the head and neck. Head, eyes, ears, nose, and throat examination did not reveal any abnormalities. Rheumatologic exam revealed deformations of the interphalangeal joints bilaterally, with fusiform enlargement of the proximal interphalangeal joints of the fingers, associated with reddish discoloration over the joints of the hands bilaterally, with no gross abnormalities of the nails. Examination of the metatarsals revealed mild erythema without other changes. Examination of knees, elbows, and other joints was unremarkable for any redness, swelling, or deformity. Cardiovascular, respiratory, neurological, and abdominal examinations were unremarkable. However, capillaroscopic examination was normal.

Upon evaluation, serologic tests including antinuclear antibody, rheumatoid factor (RF), anti-cyclic citrullinated peptide, and anti-neutrophil cytoplasmic antibody were negative. Serum levels of complements (C3 and C4) were normal. Inflammatory markers such as erythrocyte sedimentation rate and C-reactive protein were also within normal limits. Subsequent testing for plasma cryoglobulins returned positive, qualitative analysis of which revealed a faint polyclonal pattern. Investigations for potential viral etiologies, including hepatitis B and C and human immunodeficiency virus, were all negative. The total complement level (CH50) was >60 U/mL. Additional tests including serum protein and urine protein electrophoresis did not reveal a monoclonal gammopathy. Serum creatinine remained within normal limits. He was subsequently diagnosed with cryoglobulinemia, and treatment with rituximab was instituted, for which the patient received a cycle every 6 months in addition to methotrexate. He had a dramatic clinical improvement following the first cycle of therapy. He received two cycles of rituximab thus far and is currently in remission with minimal side effects of therapy.

## 3. Discussion

Cryoglobulins are single or mixed immunoglobulins that undergo reversible precipitation at low temperatures. Cryoglobulinemia is a rare illness which refers to the accumulation of cryoglobulins circulating in the blood, with the primary composition of cryoglobulin in the patient potentially determining the symptomatology [1].

Under the Brouet classification, cryoglobulinemia can be classified into three types, with type 1 as a result of monoclonal immunoglobulin, usually immunoglobulin (Ig)M, and types II and III (mixed cryoglobulinemia) containing RF IgM which complexed with the Fc portion of monoclonal IgG for type II cryoglobulinemia and polyclonal IgG in type III cryoglobulinemia [3]. Type I cryoglobulinemia is most often related to an underlying lymphoproliferative disease and may result in hyperviscosity because of the accumulation of monoclonal immunoglobulin leading to the occlusion of the microvasculature. Types II and III are most often related to chronic inflammatory states including systemic lupus erythematosus, Sjogren syndrome, and hepatitis C virus (HCV) infection [4].

Cryoglobulinemia may be also classified into primary or essential cryoglobulinemia with no known underlying cause and secondary cryoglobulinemia, occurring in association with another illness including infection, autoimmune diseases, and lymphoproliferative disorders. In terms of symptomatology, a study involving patients with type I cryoglobulinemia indicated that patients most often present with skin or vasomotor symptoms (70% of patients), in addition to nephropathy (30%) and neuropathy (47%). The underlying B cell disease was a nonmalignant monoclonal gammopathy in 36% and hematologic malignancy in 64% [5].

Most reported cases of cryoglobulinemia involve patients with underlying HCV infection, with the prevalence of mixed cryoglobulinemia which varies among countries according to the prevalence of HCV infection [6, 7]. Our case is rare in that it involves a patient who is HCV-negative with underlying juvenile idiopathic arthritis as a potential trigger. We reviewed the literature and could not find any cases reported to this day of cryoglobulinemia occurring in a patient with underlying juvenile idiopathic arthritis, which highlights the importance of assessing for cryoglobulinemia in patients who present with symptoms atypical to connective tissue disease.
